# Phase-contrast magnetic resonance imaging to assess renal perfusion: a systematic review and statement paper

**DOI:** 10.1007/s10334-019-00772-0

**Published:** 2019-08-17

**Authors:** Giulia Villa, Steffen Ringgaard, Ingo Hermann, Rebecca Noble, Paolo Brambilla, Dinah S. Khatir, Frank G. Zöllner, Susan T. Francis, Nicholas M. Selby, Andrea Remuzzi, Anna Caroli

**Affiliations:** 1 Department of Biomedical Engineering, Istituto di Ricerche Farmacologiche Mario Negri IRCCS, Bergamo, Italy; 2 MR Center, Institute of Clinical Medicine, Aarhus University, Aarhus, Denmark; 3 Computer Assisted Clinical Medicine, Medical Faculty Mannheim, Heidelberg University, Mannheim, Germany; 4 Centre for Kidney Research and Innovation, University of Nottingham, Royal Derby Hospital Campus, Nottingham, UK; 5 Department of Diagnostic Radiology, Azienda Socio-Sanitaria Territoriale Papa Giovanni XXIII, Bergamo, Italy; 6 Department of Renal Medicine, Aarhus University Hospital, Aarhus, Denmark; 7 Sir Peter Mansfield Imaging Centre, School of Physics and Astronomy, University of Nottingham, Nottingham, UK; 8 Department of Management, Information and Production Engineering, University of Bergamo, Dalmine, BG Italy

**Keywords:** Phase-contrast MRI, Renal disease, Renal blood flow, Biomarker

## Abstract

**Objective:**

Phase-contrast magnetic resonance imaging (PC-MRI) is a non-invasive method used to compute blood flow velocity and volume. This systematic review aims to discuss the current status of renal PC-MRI and provide practical recommendations which could inform future clinical studies and its adoption in clinical practice.

**Methodology:**

A comprehensive search of all the PC-MRI studies in human healthy subjects or patients related to the kidneys was performed.

**Results:**

A total of 39 studies were included in which PC-MRI was used to measure renal blood flow (RBF) alongside other derivative hemodynamic parameters. PC-MRI generally showed good correlation with gold standard methods of RBF measurement, both in vitro and in vivo, and good reproducibility. Despite PC-MRI not being routinely used in clinical practice, there are several clinical studies showing its potential to support diagnosis and monitoring of renal diseases, in particular renovascular disease, chronic kidney disease and autosomal dominant polycystic kidney disease.

**Discussion:**

Renal PC-MRI shows promise as a non-invasive technique to reliably measure RBF, both in healthy volunteers and in patients with renal disease. Future multicentric studies are needed to provide definitive normative ranges and to demonstrate the clinical potential of PC-MRI, likely as part of a multi-parametric renal MRI protocol.

**Electronic supplementary material:**

The online version of this article (10.1007/s10334-019-00772-0) contains supplementary material, which is available to authorized users.

## Introduction

The assessment of renal blood flow (RBF) is particularly important for the diagnosis and monitoring of renal diseases, including chronic and acute kidney diseases, renovascular disease, and autosomal dominant polycystic kidney disease (ADPKD), because changes in RBF are prominent at the earliest stages of disease. Different techniques have been used to determine RBF in patients with renal disease; however, these techniques may be unacceptably invasive or unable to accurately measure RBF [1]. Color Doppler ultrasonography is easily accessible, but is user dependent, and it can be technically challenging to make accurate flow measurements in overweight patients. RBF can be estimated from para-aminohippurate (PAH) renal clearance. PAH is filtered freely at the glomerulus and secreted by the tubules so that it is almost completely removed from the blood that passes through the kidneys. Therefore, the rate at which the kidneys can clear PAH (measured during a continuous infusion) reflects total renal plasma flow. However, the renal extraction rate of PAH is not 100%, meaning that the calculated ‘effective renal plasma flow’ (ERPF) tends to underestimate the true renal plasma flow. Renal extraction of PAH is usually assumed to be 85% in healthy subjects [2, 3], but can go down to 70% in patients with renal impairment [2], and the individual reduction in PAH clearance is rather unpredictable [4]. Since ERPF is calculated by dividing PAH clearance by PAH renal extraction ratio, the variability of the latter is a potential source of error [5] reducing the accuracy of the method. Phase-contrast magnetic resonance imaging (PC-MRI) is a MRI technique for determining blood flow velocity and volume in a specific vessel during the cardiac cycle. PC-MRI is already used in mainstream clinical practice in cardiology and has been extensively validated [6]. It is also reliably used in newborns [7] whose vessel size can be similar to adult renal vessels. PC-MRI, with no need for contrast agents potentially associated with risks for renal patients, provides a non-invasive alternative to measure RBF in patients.

The present article aims to systematically review all the existing literature on renal PC-MRI in healthy volunteers and patients with renal disease, to discuss the current status of renal PC-MRI as an imaging biomarker, and to provide practical recommendations which could inform future clinical studies and renal PC-MRI adoption in clinical practice.

## Methodology (inclusion and exclusion criteria)

A comprehensive search of all the PC-MRI studies in human subjects or patients related to the kidneys, excluding animal experiments, and reporting renal blood flow velocity or volume, was performed on 31 March 2019 using PubMed, and crossed checked with references cited by the related publications. Search terms are available as Online Resource (supplementary material, Section S2). A total of 54 hits were found, identifying 39 papers meeting the inclusion criteria. All the human studies involving renal PC-MRI and reporting RBF values have been summarized in tables and are available as Online Resource (see Supplementary review table).

## PC-MRI acquisition and analysis

### PC-MRI acquisition methods

PC-MRI technique is based on applying magnetic gradients such that the signal phase is made sensitive to the velocity of moving tissue or blood. This is obtained by insertion of a bipolar gradient pair between excitation and signal read-out. The velocity or flow sensitivity of the sequence is defined by the velocity encoding (Venc) parameter, which can be modified by varying the amplitude and duration of the bipolar gradient pair, and can be computed using the following formula:

$$v\mathrm{ }=\frac{\Delta\phi }{\pi } \times \mathrm{V}\mathrm{e}\mathrm{n}\mathrm{c},$$

where $\Delta \phi$ represents the phase difference. As signal phase is only unique between – $\pi$ and $+ \pi$, this corresponds to velocities being unique from – Venc to + Venc [8]. The obtained signal phase is carried over into the phase of the complex reconstructed images, and therefore, after reconstruction, two sets of images exist: the magnitude images and the velocity maps, which are the phase images.

The renal PC-MRI sequence is usually based on a 2D spoiled gradient echo pulse sequence with short repetition time (TR) and low flip angle. In general, TR and echo time (TE) should be the shortest possible to allow faster imaging and less flow induced artifacts. On a modern scanner, TE is usually below 6 ms, and TR is shorter that 13 ms. The flip angle should be low to allow rapid imaging, but is often slightly higher than the optimal flip angle, to increase inflow enhancement. Usually, in renal PC-MRI, flip angles between 10° and 30° are used. Since measurement of RBF is most commonly performed in arteries, ECG synchronization is normally applied. This can be performed either by prospective triggering or retrospective gating. The retrospective method, for which data are sampled continuously and sorted during reconstruction using the acquired ECG signal, is most commonly used currently. There have been attempts to measure renal artery flow without ECG gating [9, 10], which is faster and allows for breath-holding, but this reduces the spatial resolution or temporal sampling, degrading the accuracy and repeatability of RBF measurements [9].

PC-MRI scans are acquired as multi-phase or cine images, where a number of temporal frames during the cardiac cycle are acquired. The obtainable number of frames is inversely related to total scan time, because the number of sampled *k*-lines per frame (the turbo-factor) determines the duration for each frame. The number of frames used in renal artery blood flow measurements ranges from 15 to 80, but typically 20–30 frames are used. As flow measurements are based on the signal phase, the method is quite sensitive to ${B}_{0}$ inhomogeneity. Therefore, two segments with different velocity sensitivity are needed. These are acquired after each other, reducing the temporal resolution or increasing total scan time. The resulting velocity images are based on subtraction of the phase images from the two segments. Due to eddy-current effects and concomitant gradient fields, there might still be offset artifacts in the velocity images, i.e., zero velocity is not shown as zero. This can be corrected during post-processing by fitting a background plane to stationary regions [11]. This is still sometimes applied, but modern scanners have integrated correction methods for this error, and, therefore, offset correction should no longer be necessary [12]. When measuring blood flow in vessels which are moving significantly with respiration, such as the renal vessels, respiration control is often used. Three different strategies can be followed: making the imaging sequence so short (< 15 s) that breath-holding can be used [13]; using respiration gating [14, 15]; or making the scan time duration sufficiently long that motion-induced artifacts will partially be averaged out [16]. All three strategies have been successfully applied. The scan time for measuring in one artery, therefore, varies between 15 s [13] and 15 min [17]. Since a number of different functional MRI measurements are often included alongside PC-MRI, the scan time for the PC sequence should be kept below a few minutes. The spatial resolution needed for obtaining reasonably accurate mean flow values in vessels does not need to be too high. It has been shown that having just three pixels across the vessel diameter provides accurate blood volume flow rates [18]. With a renal artery diameter of about 5 mm, an acquired pixel size of about 1.5 mm should be sufficient. However, using a low spatial resolution may complicate the identification of the vessels during analysis, and, therefore, better spatial resolution is recommended, if possible. Typically pixel sizes between 1.0 and 1.5 mm have been used, and slice thickness of between 4 and 8 mm. Larger slice thickness would improve the signal-to-noise ratio, but at the risk of partial volume errors [19].

**Fig. 1:**
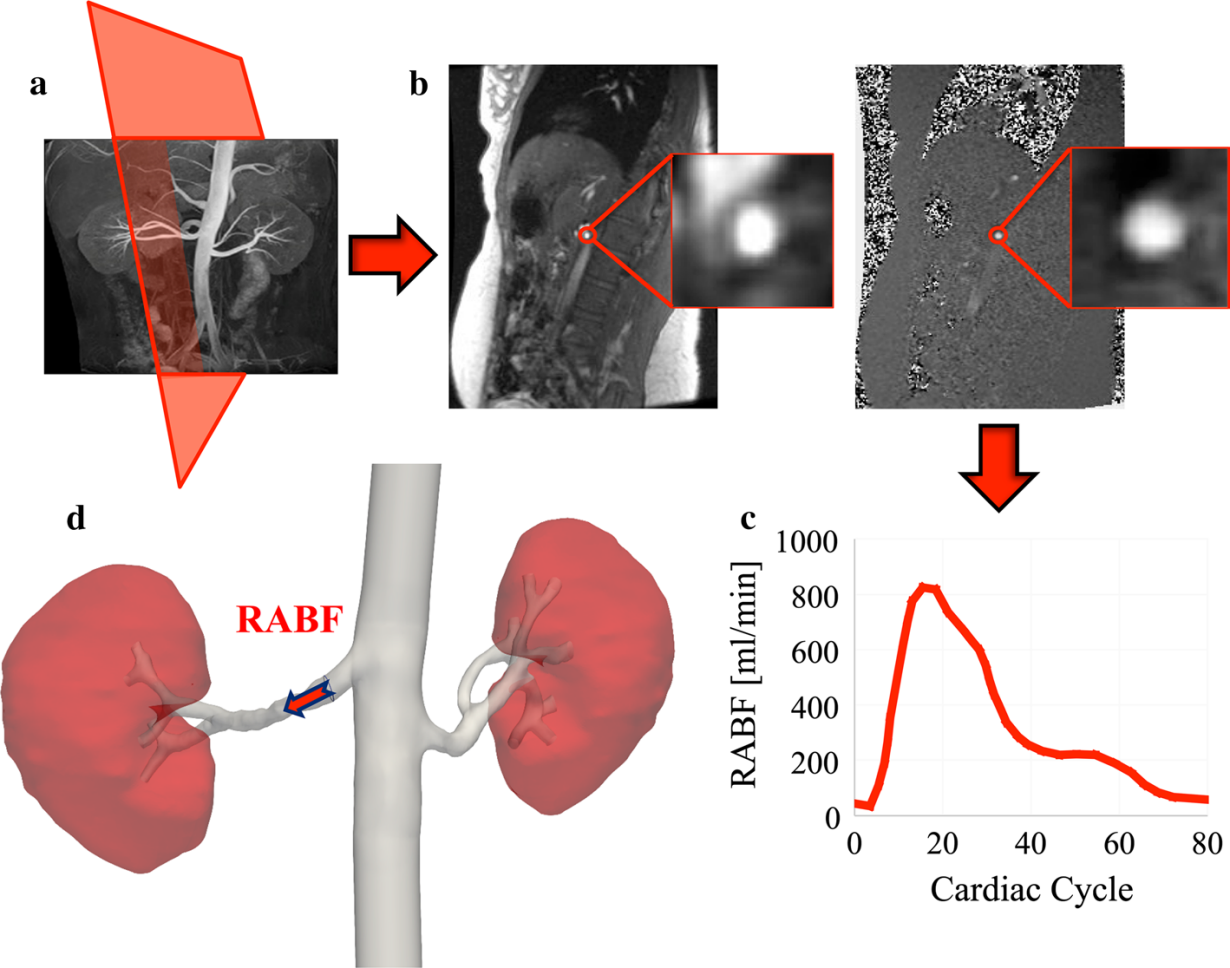
Schematic representation of phase-contrast magnetic resonance imaging (PC-MRI) acquisition and processing. **a** Prescription of PC-MRI of the right renal artery with acquisition plane perpendicular to the vessel direction. **b** Acquired coronal oblique magnitude (left) and velocity (right) images, with renal artery highlighted. **c** Profile of renal artery blood flow (RABF) in the acquisition plane defined in **a**. **d** 3D reconstruction showing average RABF computed in the right renal artery

In 2D PC-MRI, the orientation of the measurement slice should be perpendicular to the vessel direction, as only the through-plane velocity component is usually acquired (Fig. 1). For measurements in renal arteries, a good survey image, such as an angiography scan, is, therefore, strongly recommended for clear depiction of the arteries, and also to ensure that the plane is positioned prior to any bifurcations of the artery. Novel PC-MRI acquisition methods, such as the 4D flow [3D Cine PC MR angiography (MRA)] technique, make the quantification of blood flow in three dimensions possible, even during free breathing [20, 21]. The main advantage of 3D PC-MRI over 2D PC-MRI is that the former allows extracting blood velocity and flow information on any plane, rather than in a single double oblique 2D slice. Renal flow measurements can be acquired on both 1.5 T and 3.0 T MR scanners. Both field strengths are being used, and there is no clear conclusion to which is preferable. Higher field strength will add to the signal-to-noise ratio, but artifacts, such as offset errors, might worsen. In most articles, the applied Venc is around 100 cm/s. It should be higher than the peak velocity to avoid aliasing, but not much higher as this will compromise the signal-to-noise ratio. Please refer to Table 1 for a summary of practical recommendations.

**Table 1:** Recommendations for accurately measuring renal blood flow by phase-contrast MRI

**Patient preparation**	
Hydration	Potential confounder Control by hydrating the patient whenever possible
**Data acquisition**	
Slice orientation	Perpendicular to the vessel direction, prior to any bifurcations Good survey scan (e.g., angiography) needed
TR, TE (ms)	Minimum to reduce acquisition time and flow-induced artifacts
Flip angle (°)	Low to reduce acquisition time, but slightly higher than the optimal flip angle to increase inflow enhancement [(10–30) range]
Velocity encoding (cm/s)	Higher than the peak velocity to avoid aliasing Low enough not to compromise SNR (around 100)
Spatial resolution	Not too high-few pixels are enough Not too low to reliably identify vessels
Motion compensation	Breath-hold or respiratory gating
Cardiac gating	Recommended for arterial flow measurements Either prospective or retrospective ECG
Acquisition time	Below few minutes (to be compatible with multi-parametric MRI protocol)
**Image post-processing**	
Offset correction	Fit background plane to stationary regions (unless correction already integrated in the scanner)
ROI definition	Circular or elliptic, covering the lumen but not the vessel wall By manual, semi-automatic or automatic segmentation tools To be adjusted to account for movement of the vessels during the cardiac cycle
Quality control	Careful visual inspection Scan to be discarded in case of artifacts (even in few voxels of a single time frame)

In principle, RBF can be assessed in either renal arteries or renal veins, and the results should be comparable. However, renal vein blood flow has been measured only in a few papers [13, 19]. Measuring blood flow in the renal arteries is generally preferred, because they are easier to locate and the measurement planes are easier to position correctly. Alternatively, blood flow can be measured in the abdominal aorta before and after giving rise to renal arteries (inflow and outflow), with total RBF computed from the difference between the two aortic flows [22], based on the assumption that the inflow value should be equal to the sum of measurements of both renal arteries and outflow. The main advantages of measuring blood flow in the aorta is the easier detection of the vessel, the easier planning of the flow measurement slice, and the limited motion during breathing limiting blurring of the vessel contour [22]. Conversely, the main drawback is the sometimes difficult placement of the inflow measurement slice, due to the close proximity of renal arteries to the mesenteric artery.

### PC-MRI processing

Since blood flow measurement on PC-MRI is heavily affected by aliasing artifacts, a careful visual inspection beforehand is needed to have reliable RBF measurements. In case of artifacts, even if only in few voxels of a single time frame, the whole scan should be discarded [23].

Circular and elliptical regions of interest (ROIs) are commonly drawn on either the magnitude or the velocity images, covering the lumen but not the wall of the vessel of interest, with manual or semi-automatic methods using one of several processing software programs [9, 10, 16, 24–27]. ROIs cannot be kept constant across time frames, and should rather be adjusted in each of them, to account for movement of the vessels during the cardiac cycle, unless spatial registration was performed beforehand. Alternatively, automatic ROI segmentation techniques requiring no adjustment, such as adaptive thresholding [28], graph searching [29], active contour [30, 31], paraboloid velocity profiles [32] and *k*-mean clustering [8], show promise.

The mean blood velocity (expressed in cm/s) is usually computed as the average of the velocity images over all segmented pixels of all time frames [22, 33]. Then, the mean blood flow (*Q*, expressed in mL/min) (Fig. 1) is computed by multiplying the mean velocity $({v}_{\mathrm{m}\mathrm{e}\mathrm{a}\mathrm{n}},\mathrm{ }\mathrm{i}\mathrm{n} \; \mathrm{ }\mathrm{c}\mathrm{m}/\mathrm{s})$ by the ROI area $(A)$ and the conversion factor between seconds and minutes:

$$Q=60 \times A \times {v}_{\mathrm{m}\mathrm{e}\mathrm{a}\mathrm{n}}.$$

In case of 3D PC-MRI, 3D reconstruction modules from a number of software programs can be used to visualize streamlines, assess 3D blood velocity vectors, and compute other hemodynamic parameters [20, 21]. However, at present, despite 3D PC-MRI being possible, it still needs further refinement in spatial and temporal resolution to robustly allow for quantification.

## PC-MRI biomarkers

### Discovery

The feasibility of PC-MRI to measure blood flow in renal vessels, using acquisition and processing methods described above, has been well demonstrated in a number of clinical studies (see Supplementary review table, Tables 2 and 3). In 1992, Sommer et al. first showed the promise of PC-MRI to reproducibly measure blood flow in renal arteries and veins [19]. Since then, a large body of studies have used PC-MRI to measure RBF, alongside a number of derivative biomarkers: renal plasma flow (RPF), computed as the product of RBF times (1-hematocrit); renal vascular resistance (RVR), computed as ratio between the mean arterial pressure (MAP) and RBF, or ratio between (mean peritubular capillary − renal venous pressure) and afferent RBF [15, 25, 34–36]; resistive index (RI), computed as [peak systolic (PSV) − minimum diastolic velocity (MDV)]/PSV [12, 34]; renal blood flow index (RBFI), computed as RBF normalized to body surface area [37]; filtration fraction (FF), computed as percentage of the ratio between creatinine clearance and RPF [17, 25, 38]; pulsatility index (PI), computed as (PSV − MDV)/MDV [12]. An additional biomarker which could be computed from PC-MRI is the systemic vascular resistance index (SVRI), defined as (MAP − central venous pressure)/cardiac index [38, 39].

**Table 2:** Variability of renal blood flow due to different phase-contrast MRI acquisition protocols used across studies in adult healthy volunteers

Study	Sample size (F/M)	Age (years)	MR scanner	PC sequence	Respiratory gating	Cardiac gating	Velocity encoding (cm/s)	Renal blood flow (mL/min)
Sommer et al. [19]	1/8	[26–68]	NA	Cine	BELT	ECG	100	1370 ± 66 (RAtot)
Wolf et al. [52]	nTot = 10	38 [24–56]	1.5 T GE	Cine (2D)	BELT	NA	100/150	1140 ± 360 (RAtot, Venc = 100); 1108 ± 261 (RAtot, Venc = 150); 991 ± 350 (A, Venc = 150)
Maier et al. [17]	1/5	39 [25–77]	1.5 T Philips	velocity maps	FB	Prospective ECG	NA	1428 ± 540 (RAtot); 1320 ± 330 (A)
Thomsen et al. [56]	1/5	25 [21–28]	1.5 T Siemens	Segmented *k*-space	BH/FB	ECG	100	645 ± 80 (RAleft, BH); 608 ± 87 (RAleft, FB)
Schoenberg et al. [24]	nTot = 3	27 ± 6	1.5 T Siemens	Cine	BELT	ECG	150	642 ± 190 (RA)
Bock et al. [67]	nTot = 10	NA	1.5 T Siemens	IGEPI Cine	BH	ECG	70	717 ± 160 (RAleft)
Sommer et al. [51]	11/7	28 [23–45]	1.5 T GE	a. Cine TRIADS; b. Cine rapid acquisition; c. Cine non-BH rapid acquisition	BH (a, b); BELT (c)	ECG	100	1056 ± 201 (RA, a); 938 ± 143 (RA, b); 988 ± 196 (RA, c)
Michaely et al. [60]	14/10	[23–31]	1.5 T Siemens	Cine FLASH	NA	ECG	NA	506 ± 158 (RA)
Bax et al. [22]	22/19	> 40	1.5 T Philips	Cine	FB	Retrospective ECG	120	838 ± 244 (RAtot)
Park et al. [9]	2/9	[24–30]	1.5 T GE	USPC	BH	NO	100	395 ± 39** (RA)
Hackstein et al. [34]	4/8	25 [23–28]	1.5 T Philips	Gradient-echo (2D)	BH	ECG	150	[438–998] (RA)
Jin et al. [75]	4/4	NA	1.5 T Siemens	a. PC; b. RSG-PC (2D)	BH (a); BELT (b)	Retrospective ECG (a)	100 (a)	412 ± 119 (RA, a); 439 ± 146 (RA, b)
Dambreville et al. [41]	2/4	[24–39]	1.5 T Philips	FE-EPI	BH	Prospective ECG	100	[366–771] (RA); [957–1311] (RAtot)
Wittsack et al. [27]	4/2	31 ± 11	3 T Siemens	Gradient echo	BH	ECG	NA	[46–61]*** (RAtot)
Prowle et al. [38]	5/6	[26–52]	1.5 T Siemens	Cine	FB	ECG	100	[791–1750] (RAtot)
Wentland et al. [20]	4/6	[27 ± 2]	3 T GE	PC (2D)	BH/BELT	Prospective ECG/Retrospetive ECG	100/150	8 ± 2*** (RA, Venc = 100, BH, prospective ECG); 29 ± 7/16 ± 4*** (AS/AI, Venc = 150, BH, prospective ECG); 5 ± 2*** (RA, Venc = 150, BELT, retrospetive ECG)
Khatir et al. [15]	3/6	43 ± 12	1.5 T Siemens	PC (2D)	BELT	ECG	100	365 ± 119 (RA, SCAN1); 361 ± 107 (RA, SCAN2)
Steeden et al. [10]	9/11	30* [22–46]	1.5 T Siemens	a. RAGS; b. Cine	BH (a); BELT (b)	ECG (b)	100	490 ± 130 (RA, a); 480 ± 130 (RA, b)
Keegan et al. [12]	2/8	[34–37]	3 T Siemens	Spiral velocity mapping	BH	Retrospective ECG	150	413 ± 122 (RA)
Khatir et al. [59]	nTot = 24	61 ± 12	1.5 T Siemens	Gradient echo (2D)	BELT	ECG	100	[404–481] (RA)
Van der Bel et al. [39]	2/5	20 ± 1	3 T Philips	PC (3D)	NA	ECG	100	1171 (RAtot, before Ang-II); 1241 (RAtot, after Ang-II)
Cox et al. [13]	87/40	[21–69]	3 T Philips	PC	BH	ECG	100	427 ± 117 (RA, < 40 age); 329 ± 69 (RA, > 40 age)
Kline et al. [37]	6/4	22 ± 3	3 T GE	Cine (2D)	NA	NA	100	1083 ± 157 (RAtot)
Van der Bel et al. [36]	3/5	[19–31]	3 T Philips	PC (3D)	FB	ECG	100	1152 ± 75 (RAtot, baseline); 1039 ± 72 (RAtot, LBNP − 15 mmHg); 950 ± 63 (RAtot, LBNP − 30 mmHg); 1012 ± 68 (RAtot, recovery)
Eckerbom et al. [75]	15/13	23 ± 5	3 T Philips	Turbo field-echo	BH	ECG	NA	467 ± 155 (W, RAright); 453 ± 166 (W, RAleft); 543 ± 169 (M, RAright) 564 ± 78 (M, RAleft)

Age is expressed as mean ± SD or mean [range]. Only clinical studies performed on at least two healthy volunteers, and with reported renal blood flow values, are included in the table

PC, phase contrast; nTot, total number of patients (no female/male numbers available); BH, breath-holding; FB, free breathing; RA, flow computed on single renal artery; RAtot, sum of blood flows computed in right and left renal arteries; RAleft, flow computed on left renal artery; RAright, flow computed on right renal artery; AS, flow computed on superior aorta; AI, flow computed on inferior aorta; Venc, velocity encoding; LBNP, lower body negative pressure; Ang-II, angiotensin II

*Values expressed as median

**Values reported in mL/min/body surface area

***Renal artery velocity values reported in cm/s

****Values reported in mL/cardiac cycle

**Table 3:** Variability of renal blood flow due to different phase-contrast MRI acquisition protocols used across studies in adult patients with renal disease

Disease group	Study	Sample size (F/M)	Age (years)	Disease comments	Renal function (mL/min)	MR scanner	PC sequence	Respiratory gating	Cardiac gating	Velocity encoding (cm/s)	Renal blood flow (mL/min)
CKD	Debatin et al. [49]	5/3	40 [24–60]	Membranous glomerulonephritis/systemic lupus erythematosus/focal sclerosis	mGFR > 75^a^	1.5 T GE	a. 2D Cine; PC b. Six-frame TRIADS (2D); c. Conventional cine	BH (a, b); FB (c)	NA	100	821 ± 122 (RAtot, a); 953 ± 67 (RAtot, b); 1125 ± 317 (RAtot, c)
	Khatir et al. [15]	11/1	65 ± 15	CKD (stage G3–4)	eGFR < 60 (mean = 29)	1.5 T Siemens	PC (2D)	BELT	ECG	100	170 ± 130 (RA, SCAN1); 186 ± 137 (RA, SCAN2)
	Khatir et al. [58]	17/45	61 ± 13	CKD (stage G3–4)	mGFR = 36 ± 15; eGFR = 34 ± 11^a^	1.5 T Siemens	Gradient echo (2D)	BELT	ECG	100	[293–345] (RA)
	Cox et al. [13]	3/8	52 ± 14	CKD (stage G3–4)	eGFR = [15–66]^a^	3 T Philips	PC	BH	ECG	100	314 ± 148 (RA)
	Khatir et al. [60]	a.11/31; b.12/28	62 ± 2 (a); 60 ± 2 (b)	CKD (stage G3–4)	mGFR = 36 ± 22^a^ (a); eGFR = [15–60]^a^ (a); mGFR = 37 ± 3^a^ (b); eGFR = [15–60]^a^ (b)	1.5 T Siemens	Gradient echo (2D)	BELT	ECG	100	[272–399] (RA, a, AVT); [295–376] (RA, a, AVT after 18 months); [293–371] (RA, b, AnonVT); [325–430] (RA, b, AnonVT after 18 months)
	Cortsen et al. [48]	3/6	59 [49–73]	Nephrosclerosis (*n* = 3), chronic glomerulonephritis (*n* = 2), chronic nephropathy (*n* = 2), chronic pyelonephritis (*n* = l) and ADPKD (*n* = l)	eGFR = 35 ± 17 [9–57]	1.5 T Siemens	Velocity mapping	BELT	ECG	NA	691 ± 421 (RAtot)
ADPKD	King et al. [34]	81/46	32 ± 8	ADPKD	mGFR = 99 ± 23; eGFR > 70	1.5 T GE	Fast gradient echo (2D)	NA	Retrospective ECG	100	399 ± 124^a^ (RAright); 355 ± 126^a^ (RAleft)
	Torres et al. [25]	49/82	31 [15–46]	ADPKD	mGFR = 99 ± 2^a^; eGFR = 85 ± 2^a^	1.5 T GE + Philips	Fast gradient echo (2D)	NA	Retrospective ECG	100	737 ± 20^a^ (RAtot, baseline); 684 ± 16^a^ (RAtot, 1 year); 644 ± 18^a^ (RAtot, 2 year); 603 ± 23^a^ (RAtot, 3 year)
	Irazabal et al. [69]	10/10	47 ± 9	ADPKD	mGFR = 69 ± 35; eGFR ≥ 30	1.5 T GE	Fast gradient echo (2D)	NA	ECG	100	526 ± 266^a^ (RAtot)
	Spithoven et al. [41]	53/38	39 ± 11	ADPKD	eGFR = 79 ± 29^a^	1.5 T Siemens	Fast gradient echo (2D)	NA	Retrospective ECG	100	814 ± 302^a^ (RAtot)
	Kline et al. [36]	6/4	23 ± 3	ADPKD	eGFR = 111 ± 12^a^	3 T GE	Cine (2D)	NA	NA	100	1045 ± 139 (RAtot)
Renovascular disease	Schoenberg et al. [24]	11/12	57 ± 23	RAS (atherosclerosis 70%; FMD 9%; other 21%)	NA	1.5 T Siemens	Cine	BELT	ECG	150	286 ± 39 (RA, preoperative); 567 ± 106 (RA, postoperative)
	Bock et al. [67]	nTot = 15	NA	RAS (cause not stated)	mGFR > 70	1.5 T Siemens	Cine	FB	NA	NA	464 ± 188 (RAleft)
	Maier et al. [17]	0/3	[25–55]	1 × arteriosclerotic renal artery stenosis; 1 × FMD; 1 × agenesis of the right kidney	NA	1.5 T Philips	velocity mapping	FB	Prospective ECG	NA	1332 (RAtot of FMD); 1128 (RAtot of arteriosclerotic stenosis); 1044 (RAright of agenesis)
	Binkert et al. [64]	27/38	60 [24–83]	Atherosclerotic RAS (91%); FMD (9%)	NA	1.5 T GE	Fastcard	BH	Prospective ECG	80	92 ± 33 (RA)
	Binkert et al. [63]	10/13	64 [47–82]	Atherosclerotic RAS (70%); FMD (30%)	NA	1.5 T GE	Cine	BH	ECG	80	0.90 ± 0.45^b^ (RA, benefit of PTRA); 1.17 ± 0.62^b^ (RA, no benefit of PTRA)
Mixed disease group	Michaely et al. [59]	20/26	[42–68]	Renovascular disease only (37%); parenchymal disease only (22%); combined renovascular and parenchymal disease (41%)	NA	1.5 T Siemens	Cine FLASH	NA	ECG	NA	256 ± 136 (RA)
Acute kidney injury	Prowle et al. [37]	2/8	[39–74]	Sepsis-associated AKI	9/10 patients receiving CVVH	1.5 T Siemens	Cine	FB	ECG	100	[335–1137] (RAtot)

Age is expressed as mean ± SD or mean [range]. Only clinical studies performed on at least two patients, and with reported renal blood flow values, are included in the table

nTot, total number of patients (no female/male numbers available); mGFR, measured glomerular filtration rate; eGFR, estimated glomerular filtration rate; PC, phase contrast; BH, breath-holding; FB, free breathing; CKD, chronic kidney disease; ADPKD**,** autosomal dominant polycystic kidney disease; RA, flow computed on single renal artery; RAtot, sum of blood flows computed in right and left renal arteries; RAleft, flow computed on left renal artery; RAright, flow computed on right renal artery; PTRA, percutaneous transluminal renal angioplasty; FMD, fibromuscular dysplasia; AVT, vasodilating medical therapy; AnonVT, nonvasodilating medical therapy; CVVH, continuous veno-venous hemofiltration; RAS, renal artery stenosis

^a^Values reported in mL/min/1.73 m^2^

^b^Values reported in mL/min/cm^3^

### Technical validation

Technical validation of quantitative PC-MRI has been performed by comparison with alternative velocimetry/flow measurement techniques both in vitro and in vivo (see Table 4).

**Table 4:** Phase contrast MRI accuracy in quantifying renal blood flow

Study	Methods	Reference technique	Accuracy results
Ku et al. [47]	In vitro	LDV, theoretical calculations	Excellent accuracy, good correlation (no reported values)
	Recirculating steady flow phantom (25.4 mm diameter)		
Debatin et al. [50]	In vitro	True flow	Flow error: 42.1 ± 10.3% (16-frame cine PC-MRI), − 10.4 ± 17.3% (1-frame 2D PC-MRI), − 2.4 ± 2.5% (6-frame triads PC-MRI)
	Flow phantom		
Siegel et al. [45]	In vitro	LDV	Velocity error < 30% (provided high SNR, low TE, thick slices)
	Stenotic flow phantom		
Lee et al. [44]	In vitro	Doppler US	Peak systolic velocity: SEE = 5.3 cm/s (fast PC vs Doppler US), 4.6 cm/s (cine PC vs Doppler US); minimum diastolic velocity: *r* = 0.74 (fast PC vs Doppler US)
	Flow phantoms (10 mm diameter) with 0, 50, 75% stenosis		
Hoppe et al. [43]	In vitro	Doppler US guidewire	Flow: *r* = 0.95
	Flow phantoms with varying concentric stenosis		
King et al. [35]	In vitro	True flow	Steady flow: *R*^2^ = (0.77–0.99), relative flow error = (1.5–112)% according to pixel resolution [(0.8–3.6) mm]; pulsatile flow: error = (0.6–4.1)% (5 mm diameter) (1.4–13.8)% (2 mm diameter)
	Steady flow phantoms (3–11 mm diameter) + pulsatile flow phantoms (2 and 5 mm diameter)		
Hollnagel et al. [46]	In vitro	LDV, CFD	Maximum velocity: RMSE = 6.55% (internal cerebral artery, PC-MRI vs LDV), 8.02% (internal cerebral artery, PC-MRI vs CFD), 9.34% (anterior cerebral artery, PC-MRI vs LDV), 10.35% (anterior cerebral artery, PC-MRI vs CFD)
	Flow phantom		
Dambreville et al. [41]	In vitro	True flow	Steady flow: CCC = 0.992, mean flow error = 6.0 mL/min; pulsative flow: CCC = 0.996, mean flow error = − 6.7 mL/min
	Steady flow phantoms (3–11 mm diameter) + pulsatile-flow phantoms with additional 2.1 mm diameter channel		
Khodarahmi et al. [48]	In vitro	PIV	Steady flow: CC > 0.99, pulsatile flow: CC > 0.96
	Flow phantom with varying concentric stenosis		
Spithoven et al. [42]	In vitro	True flow	Flow: CC = 0.969 (6–8 mm diameter)
	Flow phantoms (5–8 mm diameter)		
Sommer et al. [19]	In vivo	PAH clearance	RBF: *r* = 0.63 (artery), *r* = 0.76 (vein)
	9 HVs		
Lundin et al. [53]	In vivo	PAH clearance	Good agreement (no reported values)
	14 HVs		
Wolf et al. [52]	In vivo	PAH clearance	RBF error = 39 mL/min (95% CI − 100 to 177) (Venc = 100), 69 mL/min (95% CI − 31 to 169) (Venc = 150);
	10 HVs		
Debatin et al. [50]	In vivo	PAH clearance	Mean RBF error = 2.8 ± 7.1% (6-frame triads PC-MRI), 28.5 ± 28.2% (16-frame), − 11.6 ± 14.9% (1-frame)
	8 HVs		
Myers et al. [1]	In vivo	PAH clearance	RBF error = 20 mL/min (95% CI − 214 to 254), *r* = 0.91
	14 renal transplant recipients		
Cortsen et al. [49]	In vivo	PAH clearance, 99mTc-DTPA scintigraphy	RBF: *r* = 0.68
	8 CKD patients		
Sommer et al. [51]	In vivo	PAH clearance	Mean RBF error = (0–95) mL/min, RBF bias = (− 1.3 to 10)%, 95% CI = ± (17.6–26.5)%, based on cine PC-MRI sequences (segmented *k* space, rapid spiral)
	18 HVs		
de Haan et al. [55]	In vivo	^133^Xenon Washout	RBF: *r* = 0.69; CC = 0.51
	71 kidneys from patients with suspected renovascular hypertension		
Spithoven et al. [42]	In vivo	Hip clearance	RBF: *r* = 0.81
	21 ADPKD patients		

PC, phase contrast; Venc, velocity encoding; PIV, particle image velocimetry; Hip, ^131^I-hippuran; SEE, standard error of the estimate; US, ultrasound; SNR, signal-to-noise ratio; ICC, intraclass correlation coefficient; RMSE, root mean squared error; CFD, computational fluid dynamics; LDV, laser Doppler velocimetry; RBF, renal blood flow; HVs, healthy volunteers; ADPKD, autosomal dominant polycystic kidney disease; CKD, chronic kidney disease; PAH, para-aminohippurate; CV, coefficient of variation; CI, confidence interval; TE, echo time; CCC*,* concordance correlation coefficient

*Comparison of PC-MRI against fluid collection in phantoms of variable diameter* Three studies have assessed PC-MRI flow measures in phantoms with a range of diameters to mimic the renal artery. King et al. [34] assessed RBF in a flow phantom of diameters of 2 and 5 mm with pulsatile flow. They showed PC-MRI accuracy was strongly related to phase-encoding pixel resolution, but when optimized, accuracy was excellent with errors in flow of < 1.4%. Dambreville et al. [40] performed prospective and retrospective gated PC-MRI in phantom studies, validating steady state and pulsatile PC-MRI flow measures against fluid collection. Results showed good accuracy, with deviations from true flow consistently below 13% for vessel diameters of 3 mm and above. Spithoven et al. [41] validated PC-MRI RBF measurements using flexible silicon phantoms of 5, 6, 7 and 8 mm renal artery diameter and 40% glycerol/60% purified water to mimic blood. RBF determined simultaneously with PC-MRI and fluid collection showed excellent agreement [correlation coefficient (*r*) of 0.97 (*p* < 0.001)].

*Comparison of PC-MRI flow against ultrasound in vitro* There are six studies using ultrasound measures as a reference technique for in vitro flow measurements, all of which show a high degree of correlation. Hoppe et al. [42] showed a good correlation (*r* = 0.95) between PC-MRI flow measurements in varying concentric stenosis with invasive Doppler guidewire measurements. PC-MRI velocity has been shown to be more accurate than Doppler ultrasound [43], suggested to arise from the fact that ultrasound only measures flow velocities along the axis of the ultrasonic beam. Laser Doppler velocimetry, which measures the velocity component of a single particle at a “given point” perpendicular to the axis of the light beam, has demonstrated a wide range of accuracy for PC-MRI for both steady state and pulsatile flows [44–46]. Particle image velocimetry, which concurrently acquires 2D velocity information, has been used to validate PC-MRI flow through stenotic phantoms with various degrees of narrowing (*r* > 0.99 and > 0.96 for steady and pulsatile flows) [47].

*Comparison of in vivo PC-MRI flow measures with alternative methods* In vivo measures of RBF measured using PC-MRI have been shown to yield a good correlation with “gold standard” methods of RBF measurement, including PAH clearance [41, 48–53], Doppler ultrasound flow probe measurements [53], 99mTc-DTPA scintigraphy [48] or ^133^Xenon washout flow measurements [54] (see “Biological validation”). Studies have developed and validated more advanced in vivo PC-MRI RBF measures. For example, Thomsen et al. [55] validated segmented *k*-space velocity mapping against conventional ECG-triggered PC-MRI, whilst Sommer et al. [50] compared spiral PC-MRI techniques, showing a range of agreement of ± 17.6% to ± 26.5%.

*Reproducibility of in vivo PC-MRI flow measures* Nine studies have shown good reproducibility and low intra- and inter-observer coefficient of variation (CV) of PC-MRI RBF measures, as summarized in Table 5. Keegan et al. [12] showed high reproducibility and low inter-observer variability of interleaved spiral phase velocity mapping for measurement of RBF and renal pulsatility indices. Abdominal 2D and 4D phase-contrast MR flow measurements have demonstrated strong repeatability and internal consistency of flow measurements [20]. Bax et al. [22] repeated PC-MRI measures in HVs as two successive scans or two scans collected at an interval of 7–21 days to differentiate between biological variation and measurement error. The mean total RBF of the HV kidney was 838 mL/min ± 244 (SD), whilst the CV was only slightly lower (17%) for two successive scans as opposed to the longer time interval (23%). A similar study assessed mean RBF and CV between two study days 1–2 weeks apart for HVs and chronic kidney disease (CKD) patients [15]. HVs and CKD patients had a single kidney RBF of 365 ± 119 mL/min and 170 ± 130 mL/min, with CVs of 8.3% and 12.9%, respectively. Dambreville et al. [40] demonstrated that breath-hold PC-MRI schemes show good week-to-week reproducibility, with a CV of 10.6%. Spithoven et al. [41] assessed PC-MRI flow measures performed three times in 21 ADPKD patients by two research physicians and showed an average intra-observer CV of 1.3% and inter-observer CV of 2.5% [34]. Both Cox et al. [13] and Kline et al. [36] measured the intra-subject repeatability of PC-MRI RBF as part of a multi-parametric renal MRI protocol, and reported a CV of 10.1% and 14.4%, respectively.

**Table 5:** Phase-contrast MRI of the renal arteries: inter-study, intra-observer and inter-observer reproducibility

Study	Methods	Reproducibility results
King et al. [34]	Repeated measurements	Intra-observer: CV = 1.2% and 1.4%, ICC = 0.987 and 0.983; inter-observer: CV = 2.5%, reliability coefficient = 0.983
	19 ADPKD patients	
Bax et al. [22]	3 repeated scans (2 successive + 7–21 days apart)	Inter-study CV = 17% (successive scans) and 23% (longer time interval)
	19 HVs	
Dambreville et al. [40]	6 repeated scans (2 successive + 4 ones 1-week apart)	Inter-study RBF difference = 30.8 ± 3.5 mL/min (successive scans); CV = 10.6% (overall), CV = 9.0% (weekly interval), CV = 4.2% (successive scans)
	6 HVs	
Wentland et al. [20]	2 repeated scans (*n* = 2)	Inter-study RBF difference = 14.0 ± 12.5% (2D PC-MRI), 15.1 ± 15.6% (4D PC-MRI)
	10 HVs	
Khatir et al. [15]	2 repeated scans (1–2 weeks apart)	Inter-study CV = 8.3% (HVs) and 12.9% (CKD); ICC = 0.92 (HVs) and 0.78 (CKD)
	11 HVs and 9 CKD patients	
Keegan et al. [12]	2 repeated scans + repeated measurements	RBF difference: 38.5 ± 20.0 mL/min (inter-observer), 17.9 ± 44.8 (inter-study, observer 1), 24.2 ± 59.0 (inter-study, observe* R*^2^)
	10 HVs	
Spithoven et al. [41]	Repeated measurements	Intra-observer: CV = 2.3%, ICC = 0.997 and 0.995; inter-observer: CV = 3.5%, ICC = 0.991
	21 ADPKD patients	
Cox et al. [13]	2–3 repeated scans	Inter-study CV = 14.4 ± 4.3%, ICC = 0.844
	11 HVs	
Kline et al. [36]	2 repeated scans (24–210 h apart)	Inter-study reproducibility: 10.1 ± 7.8%
	10 HVs	

PC, phase contrast; ICC, intraclass correlation coefficient; RBF, renal blood flow; HVs, healthy volunteers; ADPKD, autosomal dominant polycystic kidney disease; CKD, chronic kidney disease; CV, coefficient of variation*;* CCC*,* concordance correlation coefficient

### Biological validation

There are ten studies reporting comparisons of PC-MRI renal artery flow measurements against alternative techniques in humans. Of these, seven studies compared PC-MRI measurements against PAH clearance that, despite representing a suboptimal reference comparator [5], was used to estimate either ERPF or to calculate RBF from ERPF using the following equation: RBF = ERPF/(1 − hematocrit) [1, 19, 38, 48–51] (Table 4). A further three studies compared PC-MRI of renal artery flow with the difference in aortic flow (also measured by PC-MRI) above and below the renal arteries [14, 22, 52]. However, as this approach is making comparisons against the same measurement technique, it is best considered an assessment of internal validity. Finally, two of the included studies incorporated experimental arms in which PC-MRI was compared with direct measures of vessel flow. Schoenberg et al. obtained in vivo reference measurements using a transit-time US flow probe surgically implanted in the left renal artery of seven dogs [24] and using the same US method, Debatin compared measures of flow in an ex vivo phantom constructed using a 6-cm segment of human renal artery [49].

*Comparisons against PAH clearance *All studies were small in size (range 8–14 participants); five were undertaken in HVs [19, 38, 49–51], one in people with CKD [48] and one in renal transplant recipients [1]. Direct comparisons between studies are difficult as a variety of methods for PC acquisition were used, and in two studies more than one method were reported [14, 49, 50]. In addition, there was heterogeneity across studies in whether ERPF or RBF values were used as the comparator and in statistical analysis approaches. Three of the studies in HVs reported good agreement between PC-MRI renal artery flow and PAH methods. Debatin reported that the best of three different PC-MRI techniques studied had a low mean difference of 2.8 ± 7.1% versus PAH RPF [49]. In a similar study that also assessed different PC-MRI methods, Sommer et al. found mean differences that ranged from 0 mL/min (95% CI − 166 to 166 mL/min) at best to 95 mL/min (95% CI − 154 to 341 mL/min) [50]. Wolf et al. also reported good agreement [39 mL/min mean difference (95% CI − 100 to 177 mL/min)], although results differed slightly depending on velocity encoding [51]. A single study in nine participants reported better correlations between PC-MRI measures of renal vein flow and PAH as compared to arterial flow [50]; this informed a follow-on study in 14 renal transplant recipients with preserved renal function that found both good correlation (*r* = 0.92) and low mean difference (20 mL/min, 95% CI − 214 to 254 mL/min) between the two techniques [1]. The only study that included participants with reduced estimated glomerular filtration rate (eGFR) showed a reasonable correlation between PAH and PC-MRI measures of RBF, noting that only eight patients were studied and PAH may be less accurate if tubular secretion is impaired [48]. Finally, Van der Bel et al. reported changes in PC-MRI renal artery blood flow and ERPF in response to angiotensin infusion in 8 HVs and showed similar patterns of change but did not directly compare the two measurement techniques [38].

*Comparison against aortic inflow/outflow* Three studies compared direct measures of renal artery flow by PC-MRI against difference in PC-MRI measures of aortic flow above and below the renal arteries [14, 22, 52]. In 18 HVs, Bax et al. reported a reasonable correlation between the two (*r* = 0.72, *p* = 0.002) [22]. De Haan showed a similar correlation in a graphical figure without reporting values [14], whilst Lundin reported no significant difference in mean total RBF calculated from the sum of the renal artery flows (RAs) versus flow calculated from the aortic difference (mean ratio RBF: aortic difference 1.06 ± 0.04, range 0.79–1.20) [52].

*Experimental studies* Whilst this review was restricted to studies in humans, two studies included experimental arms. Schoenberg reported that the relative accuracy of mean flow measured by PC-MRI was within 4.1 ± 2.9% of that measured by transit time ultrasound in the left renal artery of seven dogs [24]. In an ex vivo phantom built with a human renal artery, Debatin found a range of mean differences (42.1 ± 10%, − 10.4 ± 17.3% and − 2.4 ± 2.5%) across three PC-MRI methodologies [49]. Notably, the method that performed best in this study was shown to have the greatest bias in the study of Sommer comparing against PAH clearance in vivo [50].

## PC-MRI clinical application in renal disease

### Chronic kidney disease

In patients with chronic kidney disease (CKD), structural changes, including reduction in total nephron number, interstitial fibrosis, and/or vascular rarefication [56] often develop before measurable functional changes [57]. Structural changes may be associated with multiple systemic diseases, for instance diabetes mellitus, hypertension, and arteriosclerosis, and may in turn affect RBF, likely reducing it due to the increased resistance of renal microcirculation. Moreover, while in healthy subjects the kidney is very effective in regulating blood flow over a wide range of blood pressures and in maintaining glomerular pressure and filtration rate, in patients with CKD this autoregulation may be gradually lost, as renal function declines, resulting in RBF decrease. In addition, CKD patients receive a wide range of drugs, including diuretics and renin–angiotensin system inhibitors, which may alter renal function and blood flow and influence renal hemodynamics. There are only few studies using PC-MRI in CKD (Table 3), and the method is currently not used routinely to assess RBF in patients with CKD in the clinic. A good reproducibility of respiratory-gated PC-MRI was identified in CKD patients and HVs, when examined 1–2 weeks apart, revealing coefficients of variation of 12.9% and 8.3%, respectively [15]. RBF measured by PC-MRI was significantly decreased in CKD patients compared to HVs [15, 48, 58], even in patients with mild-to-moderate CKD, although the HVs were 8 years younger [13]. Combining PC-MRI and arterial spin labeling (ASL), Michaely et al. were able to separate healthy kidneys from kidneys with vascular, parenchymal or combined disease [59]. In patients with CKD, measured GFR was reduced to a greater extent than RBF, resulting in a reduced filtration fraction, which may reflect an adaptation to keep intra-renal oxygenation within normal range [58]. Last, PC-MRI was used to measure renal arterial blood flow and calculate renal vascular resistance in a study comparing vasodilatory and non-vasodilatory antihypertensive treatment in patients with CKD. After 18-month follow-up, RABF increased significantly in both groups, but the change did not differ between groups [60].

### Acute kidney injury

Changes in RBF and/or perfusion are considered critical to the etiology of many forms of acute kidney injury (AKI). However, PC-MRI has only rarely been applied in people with AKI, which may in part reflect the perceived logistical difficulties of scanning acutely unwell patients. Following an initial report of feasibility [39], Prowle et al. have shown that it is possible to successfully perform PC-MRI in intensive care unit (ICU) patients with sepsis-associated AKI. In a pilot study of 10 people, of whom 8 were mechanically ventilated, 9 were on continuous hemofiltration and 5 required vasopressors, RBF and cardiac output (CO) were measured and compared with 11 HVs [37]. Results, which should be regarded as exploratory, showed that median RBF in septic AKI (482 mL/min) was lower than that in healthy controls (1260 mL/min); that there was considerable variation in RBF measures (range 335–1137 mL/min in AKI group); and that RBF as a proportion of CO was also reduced (suggesting a dependency of RBF on changes in CO).

### Renovascular disease/renal artery stenosis

Renal artery stenosis (RAS) is a leading cause of secondary hypertension and can cause CKD. In unselected populations, several large trials have failed to show significant benefit to intervention with angioplasty and/or stenting [61, 62]. Despite these findings, debate continues as to whether subgroups of patients with RAS may benefit from intervention, and if so how best to identify them. A small number of studies have, therefore, applied PC-MRI to patients with RAS to determine whether functional measurements of renal artery flow/velocity provide additional clinical information. Seven studies report on the use of PC-MRI in the context of renovascular disease [16, 17, 27, 59, 63–65].

Several studies evaluated whether PC-MRI can improve characterization or detection of anatomical severity of renal artery lesions (Table 3). In RAS, Cine PC-MRI demonstrates a damped systolic wave which is longer in duration [66]. Schoenberg et al. performed cardiac-gated Cine PC-MRI in 23 patients with 48 areas of RAS and MR flow measures were compared against severity of anatomical stenosis [24]. To separate those with > 50% stenosis from those with no stenosis, PC-MRI was reported to have 100% sensitivity and 93% specificity. In 11 patients, ultrasound flow measures of the renal artery were also taken at time of surgical intervention and correlated well with PC-MRI measures. Post-operative PC-MRI flow values improved, but no clinical outcomes were reported. It has also been hypothesized that intravenous angiotensin-converting enzyme inhibitor (ACEi) administration may improve diagnostic accuracy of waveform analysis, but this was not borne out in a study of 35 patients [16]. In a pilot study, Bock et al. compared two PC-MRI techniques (interleaved gradient echo-planar technique (IGEPI) Cine PC-MRI and conventional Cine PC-MRI [67]. IGEPI Cine PC-MRI detected 5/5 high-grade stenosis versus 3/5 (66%) with conventional Cine PC-MRI. Later, Schoenberg et al. demonstrated that a combined morphologic and functional MR examination significantly reduced inter-observer variability across 7 readers evaluating 43 renal arteries [65, 68]. They reported that this approach offered reliable, reproducible grading of RAS when compared with X-ray digital subtraction angiography (DSA) AND 3D gadolinium MR.

Binkert et al. combined arterial flow volume and renal volume in 130 kidneys from 65 patients in attempt to determine functional significance of RAS lesions [64]. Of 31 kidneys with RAS, 18 had significantly reduced volume [3.08 ± 0.75 (au)] and significantly reduced flow volumes (91.56 vs 279.15 mL/min without RAS). Based on the Renal Flow Index (RFI) (flow/renal volumes), there was only minimal overlap between normal volume kidneys with RAS and those without RAS suggesting that RFI could be used to predict the likelihood of hemodynamically significant RAS. The same group later went on to investigate whether this information could be used to predict positive clinical outcomes following percutaneous angioplasty [63]. In a group of 23 patients, 34 areas of RAS were present and 11 people had bilateral disease. Clinical success (defined as a fall in diastolic BP by > 15% or a fall in creatinine of > 20%) was observed in 11 patients, 10 of whom had normal kidney volume pre-intervention. The sensitivity of RFI to predict response to therapy was reasonable (91%) but specificity low, suggesting that direct translation of this method to clinical practice would result in a significant rate of unnecessary procedures. RFI < 1.5 mL/min/cm^3^ had a 100% sensitivity predicting clinical benefit, but low specificity of 33%, although combining with clinical variables improved specificity somewhat to 67%.

### Autosomal dominant polycystic kidney disease

PC-MRI has been used to non-invasively measure RBF in autosomal dominant polycystic kidney disease (ADPKD) (Table 3) since 2003 when, in a large study of 127 patients with early ADPKD, RBF was shown to have high accuracy and intra- and inter-observer reproducibility, to strongly correlate with both renal volumes and GFR, and to predict GFR [34]. In a subsequent longitudinal study by the same group including 131 patients with early ADPKD, RBF decreased over 3-year follow-up, preceding GFR decline, was negatively correlated with total kidney (TKV) and total cyst volume slopes, and positively correlated with GFR slope, predicting structural and functional disease progression and showing promise as outcome measure in clinical trials on ADPKD [25]. PC-MRI was used in a small clinical trial to investigate, alongside GFR and TKV, the short-term effects of Tolvaptan in patients with ADPKD; the study found no significant change in RBF after 1 week of Tolvaptan treatment, with PC-MRI mirroring PAH clearance flow measurements [69]. More recently, Spithoven and colleagues provided additional evidence of accuracy and validity of RBF measurement by PC-MRI as compared with RBF measured by continuous hippuran infusion, in a cohort of 91 ADPKD patients with a wide range of eGFR values. In this study, RBF values were associated with ADPKD severity, and technical problems preventing RBF measurement occurred predominantly in patients with lower eGFR (< 70 mL/min), suggesting that RBF measurement may be less feasible in patients with ADPKD at an advanced stage [41]. Last, PC-MRI was performed in a small cohort of young patients with early-stage ADPKD and normal controls, as part of a comprehensive multi-parametric renal MRI protocol. Besides its preliminary results, showing no statistically significant difference in RBF between young ADPKD patients and normal controls [36], the study represents a valuable attempt to combine PC-MRI with other quantitative renal MR techniques towards a comprehensive characterization of the ADPKD kidney tissue and function.

## Discussion

The current published literature supports PC-MRI as a feasible and valid non-invasive technique to reliably measure renal blood flow, alongside a number of derivative hemodynamic parameters, in both HVs and patients with renal disease. There are a few key recommendations (summarized in Table 1) to be followed to accurately measure RBF by PC-MRI, possibly reducing the wide variability in the measurements reported so far (Table 2). As a potential confounder, patient hydration should be controlled whenever possible. The acquisition slice should be placed perpendicularly to the vessel direction, and prior to any bifurcation; to this purpose, a good survey scan (e.g. angiography) is extremely helpful. To minimize acquisition time and flow-induced artifacts without compromising signal-to-noise ratio, relaxation and echo times should be minimum, velocity encoding should be higher than the peak velocity (around 100 cm/s), flip angle should be low [in [10°–30°] range); spatial resolution should be sufficient to enable reliable identification of the vessels. Motion compensation should be performed by breath-hold or respiratory gating, and either prospective or retrospective cardiac gating should be used, especially for arterial flow measurements. Once acquired, PC-MRI should undergo a careful visual inspection, and images with any artifact should be discarded. To quantify renal blood velocity and volume, circular or elliptic ROIs should be defined, covering the lumen but not the vessel wall. Importantly, these ROIs should be adjusted to account for movement of the vessels during the cardiac cycle, many software packages perform such automatic tracking but this should be visually checked. PC-MRI acquisition and post-processing procedures are quite straightforward with standard software, so there is no need for a high-level of technical expertise.

PC-MRI has been technically validated in a number of studies both in vitro, using flow phantoms, and in vivo, generally showing good correlation with gold-standard methods of RBF measurement. Moreover, a large number of studies have investigated the reproducibility, and intra- and inter-observer CV of RBF measures obtained by PC-MRI, showing an overall good reproducibility. PC-MRI has been biologically validated against alternative techniques in humans, especially against PAH clearance, showing an overall good agreement between PC-MRI and PAH measurements. In addition, PC-MRI has been experimentally validated against direct measures of vessel flow [24, 49], although this is out of the scope of this clinical review. Despite PC-MRI not being routinely used in clinics, there are a number of clinical studies showing its potential to support diagnosis and monitoring of renal diseases, in particular CKD, renovascular disease, and ADPKD, particularly in the earlier stages. In HVs, the variability in RBF values, both in individual studies and across studies, is rather large, making the definition of normative ranges not possible yet. Future large multicentric studies are needed to provide reliable and definitive reference ranges.

PC-MRI is likely to benefit from combination with other promising renal MRI techniques (such as BOLD [70], DWI [71], T1 and T2 mapping [72], and ASL [73]) providing complementary information on renal microstructure and function and enabling a complete assessment of the normal and diseased kidney, potentially improving renal disease diagnosis and monitoring. Multi-parametric renal MRI has been recently pioneered in patients with CKD [13] and ADPKD [36]. Future multicenter studies are needed to demonstrate the clinical potential of PC-MRI as part of a multi-parametric renal MRI protocol. International collaborative efforts such as the COST action PARENCHIMA (https://www.renalmri.org) may help in answering this need.

## Electronic supplementary material

Below is the link to the electronic supplementary material.

Supplementary file1 (PDF 59 kb)

Supplementary file2 (XLSX 44 kb)
