# Correction to: Phase-contrast magnetic resonance imaging to assess renal perfusion: a systematic review and statement paper

**DOI:** 10.1007/s10334-020-00849-1

**Published:** 2020-06-11

**Authors:** Giulia Villa, Steffen Ringgaard, Ingo Hermann, Rebecca Noble, Paolo Brambilla, Dinah S. Khatir, Frank G. Zöllner, Susan T. Francis, Nicholas M. Selby, Andrea Remuzzi, Anna Caroli

**Affiliations:** 1 Department of Biomedical Engineering, Istituto di Ricerche Farmacologiche Mario Negri IRCCS, Bergamo, Italy; 2 MR Center, Institute of Clinical Medicine, Aarhus University, Aarhus, Denmark; 3 Computer Assisted Clinical Medicine, Medical Faculty Mannheim, Heidelberg University, Mannheim, Germany; 4 Centre for Kidney Research and Innovation, University of Nottingham, Royal Derby Hospital Campus, Nottingham, UK; 5 Department of Diagnostic Radiology, Azienda Socio-Sanitaria Territoriale Papa Giovanni XXIII, Bergamo, Italy; 6 Department of Renal Medicine, Aarhus University Hospital, Aarhus, Denmark; 7 Sir Peter Mansfield Imaging Centre, School of Physics and Astronomy, University of Nottingham, Nottingham, UK; 8 Department of Management, Information and Production Engineering, University of Bergamo, Dalmine, BG Italy

## Correction to: Magnetic Resonance Materials in Physics, Biology and Medicine (2020) 33:3–21 10.1007/s10334-019-00772-0

The article Phase‑contrast magnetic resonance imaging to assess renal perfusion: a systematic review and statement paper, written by Giulia Villa, Steffen Ringgaard, Ingo Hermann, Rebecca Noble, Paolo Brambilla, Dinah S. Khatir, Frank G. Zöllner, Susan T. Francis, Nicholas M. Selby, Andrea Remuzzi and Anna Caroli, was originally published electronically on the publisher’s internet portal on 17 August 2019 without open access. With the author(s)’ decision to opt for Open Choice the copyright of the article changed on 24 April 2020 to © The Author(s) 2020 and the article is forthwith distributed under a Creative Commons Attribution 4.0 International License (https://creativecommons.org/licenses/by/4.0/), which permits use, sharing, adaptation, distribution and reproduction in any medium or format, as long as you give appropriate credit to the original author(s) and the source, provide a link to the Creative Commons licence, and indicate if changes were made.

The original article has been corrected.

