# Supplementary material for: Phase-contrast magnetic resonance imaging to assess renal perfusion: a systematic review and statement paper
Source: MAGMA. 2019 Aug 17;33(1):3–21. doi: 10.1007/s10334-019-00772-0 (PMC7210220; doi:10.1007/s10334-019-00772-0)
Supplement: Supplementary file 1 — Supplementary file1 (PDF 59 kb) [file 10334_2019_772_MOESM1_ESM.pdf]

Supplementary material to

Villa G et al.

Phase Contrast Magnetic Resonance Imaging to assess renal perfusion: a systematic review and statement paper

Magn Reson Mater Phy

S1. Abbreviations

A : area

ACEi : angiotensin converting enzyme inhibitor

ADPKD : autosomal dominant polycystic kidney disease

AKI : acute kidney injury

ASL : arterial spin labeling

CC : correlation coefficient

CV : coefficient of variation

CKD : chronic kidney disease

CO : cardiac output

DSA : digital subtraction angiography

eGFR : estimated glomerular filtration rate

ERPF : effective renal plasma flow

FF : filtration fraction

GFR : glomerular filtration rate

HVs : healthy volunteers

ICC : intraclass coefficient

ICU : intensive care unit

IGEPI : interleaved gradient echo-planar technique

LDV : laser Doppler velocimetry

MAP : mean arterial pressure

MDV : minimum diastolic velocity

MRA : MR angiography

MRI : magnetic resonance imaging

PAH : para-aminohippurate

PC : phase-contrast

PC MRA : phase-contrast magnetic resonance angiography

PC-MRI : phase-contrast magnetic resonance imaging

PI : pulsatility index

PIV : particle image velocimetry

PSV : peak systolic velocity

Q : mean blood flow

RBF : renal blood flow

RBFI : renal blood flow index

RFI : renal flow index

RI : resistive index

ROI : region of interest

RPF : renal plasma flow

RVR : renal vascular resistance

SVRI : systemic vascular resistance index

TE : echo time

TKV : total kidney volume

TR : repetition time

Venc : velocity encoding

Vmean : mean velocity

S2. Search terms

A comprehensive search for all the PC-MRI studies in human subjects or patients related to the kidneys and excluding animal experiments was performed on 31 March 2019 in PubMed using the following terms:

(((((((((((((((((((((((renal blood flow[Title/Abstract]) OR RBF[Title/Abstract]) OR RABF[Title/Abstract]) OR renal artery blood flow[Title/Abstract]) OR renal arteries blood flow[Title/Abstract]) OR renal artery flow[Title/Abstract]) OR renal arteries flow[Title/Abstract]) OR renal blood velocity[Title/Abstract]) OR renal artery blood velocity[Title/Abstract]) OR renal arteries blood velocity[Title/Abstract]) OR renal artery velocity[Title/Abstract]) OR renal arteries velocity[Title/Abstract]) OR kidney blood velocity[Title/Abstract]) OR kidney blood flow[Title/Abstract])) OR flow measurements in the renal arteries[Title/Abstract]) OR Flow imaging of renal

arteries[Title/Abstract])))) OR Flow imaging of renal arteries[Title/Abstract])) AND ((((((((((((((((((phase contrast mri[Title/Abstract]) OR pc mri[Title/Abstract]) OR phase contrast magnetic resonance imaging[Title/Abstract]) OR phase contrast sequence[Title/Abstract]) OR phase-contrast sequence[Title/Abstract]) OR velocity map[Title/Abstract]) OR velocity mapping[Title/Abstract]) OR pc-mri[Title/Abstract]) OR phase-contrast magnetic resonance imaging[Title/Abstract]) OR pc magnetic resonance imaging[Title/Abstract]) OR pc-magnetic resonance imaging[Title/Abstract]) OR phase-contrast MR imaging[Title/Abstract]) OR hase contrast (PC) magnetic resonance imaging (MRI)[Title/Abstract]) OR PCMR[Title/Abstract]) OR phase contrast flow measurements[Title/Abstract]) OR velocity-encoded MRI[Title/Abstract]) OR MR blood flow sequences[Title/Abstract]) OR phase-contrast mri[Title/Abstract])
